# On the Mal d'Aleppo

**Published:** 1814-05

**Authors:** Thomas Girdlestone

**Affiliations:** Yarmouth


*For the Medical and Physical Journal.*

*On the Mal d'Aleppo; by* Dr. GIRDLESTONE.

THE description which you have quoted from the 1st edition of Dr. Russell's Aleppo, of the Mal d'Aleppo, is nearly verbatim to that which is given in the 2d edition. But there are facts added to the 2d edition, which may be of importance to the history of this disease. I shall endeavour to reduce them into the following sentences.

As the Mal very rarely, if ever, returned in the same patient, Dr, Russell, in order to prevent it from breaking out on on the face, made some incisions on the arms and legs of those who had never had the disease, and inserted fresh matter from the Mal into those incisions. But the few trials which he made did not succeed: the wounds healed up without producing any sign of Mal. Dr. Russell, after the publication of his 1st edition, had an opportunity of verifying his former opinion, that the Mal was less apt to leave a scar, and terminated sooner, when left to Nature, than when treated by internal medicine or external applications: for a female slave, on entering the harem of a bashaw, had a tumour on her lip; and the bashaw having been a little acquainted with medical practice, would not rest till a mercurial salivation, decoction of the woods, caustics, &c. &c. &c. were tried and persisted in for some months. As the disease grew worse, all medical means were at last laid aside. The Mal then ran its usual course: so that the officiousness of the bashaw only prolonged the disease eight or nine months beyond its usual period of termination, and gave the poor girl several weeks of unnecessary pain.

THOMAS GIRDLESTONE, M.D.

*Yarmouth, April* 6, 18.4.

